# The Rare Adverse Effect of Myalgia After Dupilumab Biologic Therapy for Refractory Atopic Dermatitis

**DOI:** 10.7759/cureus.60701

**Published:** 2024-05-20

**Authors:** Brigitte L Cochran, Jonathan R Raymond-Lezman, Alexis Coican, Nathan Sagasser, Rutva Patel, Summer Batrous, Melinda F Greenfield

**Affiliations:** 1 Osteopathic Medicine, Philadelphia College of Osteopathic Medicine, Moultrie, USA; 2 Foundational Sciences, Nova Southeastern University Dr. Kiran C. Patel College of Osteopathic Medicine, Clearwater, USA; 3 Dermatology, Orange Park Medical Center, Jacksonville, USA; 4 Medicine, University of South Florida, Tampa, USA; 5 Medicine, University of Florida, Gainesville, USA; 6 Medicine, University of North Florida, Jacksonville, USA; 7 Dermatology, Advanced Dermatology and Cosmetic Surgery, Ponte Vedra Beach, USA

**Keywords:** myalgia, shave biopsy, atopic dermatitis (ad), dupilumab adverse reaction, dupilumab

## Abstract

Dupilumab, a systemic injectable biologic, can be prescribed to patients with atopic dermatitis who do not respond to topical treatments. Atopy can frequently subside by blocking inflammatory pathways, such as interleukin-4 (IL-4) and interleukin-13 (IL-13) in the immune system. Dupilumab is generally well-tolerated and mild; the most common adverse reactions listed are arthralgia, back pain, and conjunctivitis, which clears upon cessation or finalization of dupilumab therapy. This case report describes a patient experiencing severe myalgia - a rare adverse effect. The patient’s atopic dermatitis was refractory toward topical treatments, but within one month of starting dupilumab, he experienced severe myalgias and muscle spasms, which prompted cessation of dupilumab despite it working well for his atopic dermatitis.

## Introduction

Atopic dermatitis is a chronic inflammatory skin condition that affects up to 20% of the worldwide population. It is characterized by pruritic erythematous plaques and papules which can lichenify. The pruritis can be described as stinging, burning, and severe and can cause decreased quality of life. The main treatment guidelines include topical therapies, phototherapy, and systemic immunosuppressants [1].

Dupilumab (Dupixent®) is an injectable biologic approved in 2017 by the United States (US) Food and Drug Administration (FDA) for the systemic treatment of atopic dermatitis. It is typically used in patients who fail topical therapies. It exerts its effects by blocking interleukins 4 and 13 (IL-4 and IL-13). IL-4 and IL-13 drive a T-helper 2 (Th-2) response, which plays a critical role in the pathogenesis of atopic conditions like atopic dermatitis and allergies. One of the most common adverse effects of dupilumab is conjunctivitis, which usually resolves by the end of the treatment period. Arthralgias can also occur, but myalgias are rare. A US cohort study stated that myalgia only accounted for 0.7% of the notable adverse effects of dupilumab use, making it an exceedingly rare occurrence [2]. We present a case report for a patient experiencing severe myalgia located in bilateral thighs after using dupilumab. Our goal is to increase awareness of this severe but rare adverse effect and to provide clinically relevant information for providers who utilize dupilumab [2,3].

## Case presentation

**Figure 1:**
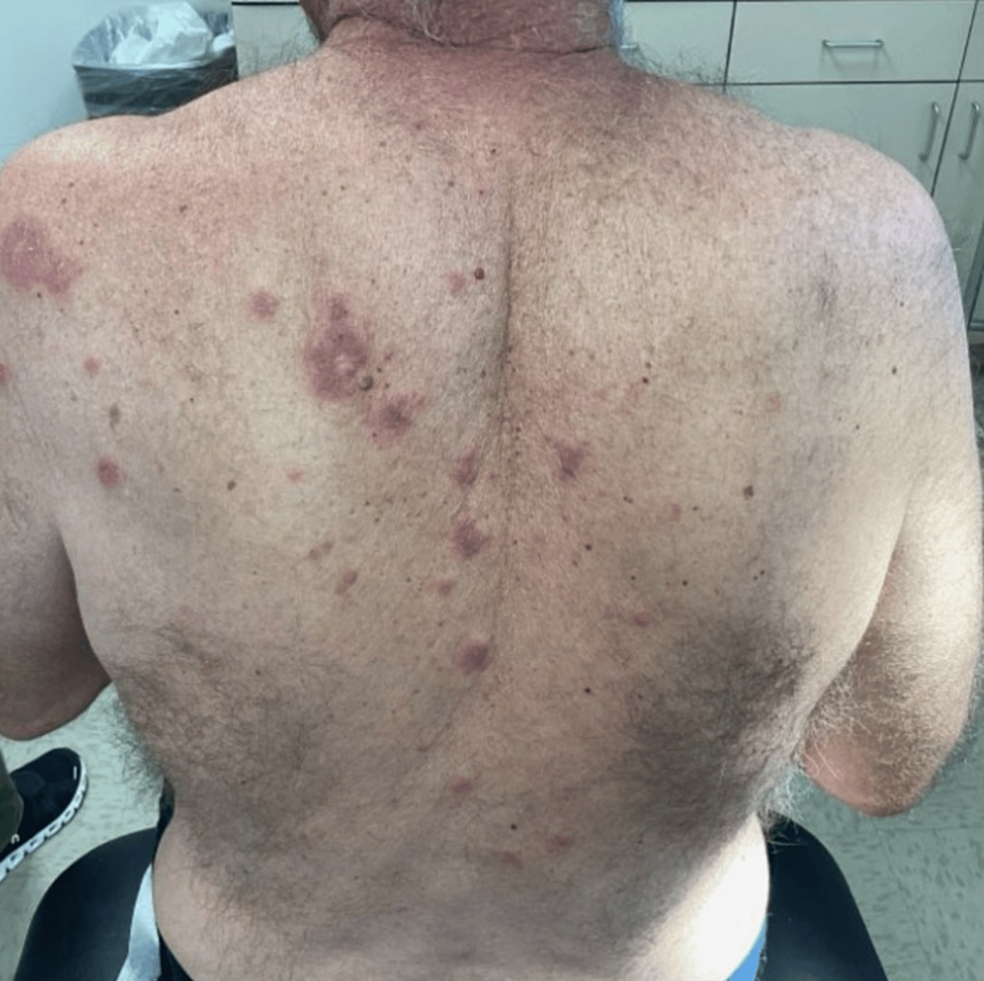
Clinical presentation of the patient The image shows erythematous patches and plaques located on the right superior back and medial and left posterior shoulder.

A 68-year-old male presented on July 3, 2023, for dermatitis unspecified located on the right superior back, medial and left posterior shoulder. The rash is described as a mixture of erythematous plaque and patches (Figure 1). He was initially seen on April 21, 2023, for a pruritic rash that covered a body surface area (BSA) of 20% and was treated initially with clobetasol 0.05% cream three times daily for five to seven days, then as needed for flares. The final diagnosis of atopic dermatitis was made when the shave biopsy was collected, and the patient was informed of the results.

Since his initial visit, the pruritic rash persisted despite treatment with high-strength topical steroids that had no improvement in his itch, as well as BSA remaining at 20%. His itch numeric rating scale (NRS) was rated 7.0 and labeled as a moderate itch that is present throughout the day and night. Due to his failure to improve with clobetasol 0.05% cream, a shave biopsy was performed to further explore the pathology of the pruritic erythematous patches to determine optimal treatment options due to the decrease in the quality of life that the patient was experiencing. Differential diagnosis of the area was dermatitis unspecified vs. psoriasis vs. nummular dermatitis vs. atopic dermatitis. The patient was given eight tablets of prednisone, 10 mg tablet, to take by mouth, once daily, with a meal in the morning. Mometasone 0.1% topical cream was prescribed to be applied twice daily for 10-14 days then as needed for flares while awaiting the pathology final diagnosis.

**Figure 2:**
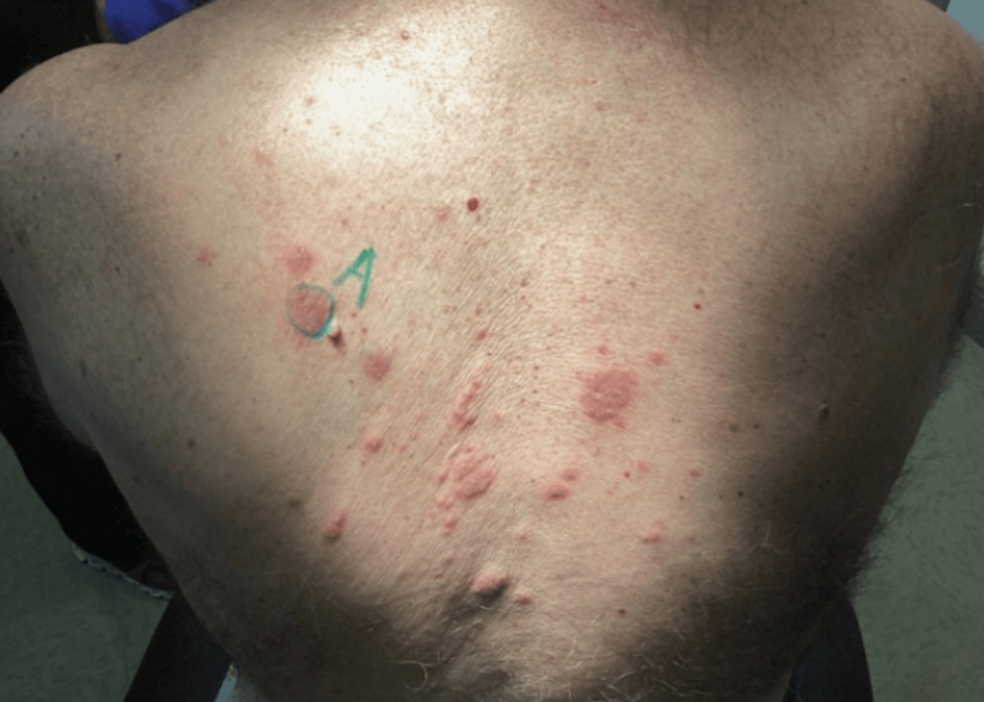
Clinical picture of the patient after the shave biopsy A shave biopsy was performed (labeled A) on an erythematous macule located on the left posterior shoulder that confirmed contact, atopic or nummular dermatitis with pathology demonstrating spongiotic dermatitis with eosinophils. Clinical correlation was recommended by pathology, which was later chosen as a final diagnosis of atopic dermatitis due to the dermatologist’s clinical diagnosis.

The shave biopsy was performed on the left posterior shoulder (Figure 2). Briefly, the area was prepped with alcohol and local anesthesia was given with 0.4 cc of 1% lidocaine with epinephrine. A biopsy was taken by the shave method to the level of the dermis. Hemostasis was obtained through the usage of aluminum chloride. The biopsy site was covered with petroleum jelly and a bandage was applied to the area.

The final diagnosis of atopic dermatitis was made when the shave biopsy was collected, and the patient was informed of the results. Considering the lack of progress from conventional treatment with a topical corticosteroid for atopic dermatitis, the healthcare practitioner concluded that the most effective course of treatment would be to initiate biologic therapy with dupilumab. This decision was based on the biologic therapy's ability to quickly clear the condition compared to topical therapy.

The biologic therapy successfully cleared the dermatitis. The patient had three doses, including an initial subcutaneous injection of 600 mg on day 1 and then a 300 mg subcutaneous injection every other week. After his third treatment dose, he returned to the office with an adverse drug reaction of severe myalgia and arthralgia. 

The patient stated that he began experiencing extreme muscle and joint pain that he had never experienced before. He described the muscle pain and/or spasms as constant pain located in his bilateral thighs that was different from the joint pain that he was experiencing in his bilateral hips and knees. He stated that this pain was impacting his sleep and daily lifestyle, now unrelated to the atopic dermatitis that was cleared with biologic therapy. The patient denied new workouts and/or trauma to his body. He stated that even though dupilumab helped resolve his atopic dermatitis pruritus, he would like to stop treatment with dupilumab as the muscle pain and/or spasms and joint pain were decreasing his quality of life.

## Discussion

Atopic dermatitis is a chronic, immune-mediated disease affecting a large number of people worldwide and has been historically treated with various topical therapies. Before dupilumab, topical corticosteroids and general skincare emollients were the most popular ways to manage atopic dermatitis. The use of systemic agents such as oral corticosteroids or immunosuppressants typically have limited use, unless the cases are showing minimal to no response to the topical agents. Additionally, long-term use of systemic agents is not recommended due to the side effects associated with the drugs [4]. Approximately 20% of those with atopic dermatitis have moderate-to-severe disease unremitting with the standard treatments [1,4]. Once standard treatments have been exhausted with little positive response, dupilumab can be prescribed to modulate symptoms from atopic dermatitis. 

Since its inception, the most common adverse effects from dupilumab included conjunctivitis and arthralgias, which typically clear after treatment resolution or cessation. This report describes a rare development of severe dupilumab-associated myalgias presented within one month of treatment. While rare adverse effects can occur, it is vital to be aware of any new symptoms patients starting dupilumab therapy may be experiencing. Furthermore, understanding the rare adverse effects that can be caused by dupilumab can ensure clinicians are focally aware of new patient complaints in follow-up appointments post-dupilumab initiation.

While myalgias are a rare occurrence of dupilumab treatment, it was substantial enough in this patient to warrant discontinuation of dupilumab despite it benefiting his atopic dermatitis exacerbation. The risks and benefits of every medication are a cornerstone for prescribing, and thoughtful conversations with patients who may have new onset symptoms should warrant a second look at the potential adverse effects of dupilumab.

## Conclusions

Due to the rare adverse drug reaction of severe myalgia, as well as a common adverse reaction of arthralgia, it is important to communicate with the patient effectively prior to the initiation of dupilumab biologic therapy. The provider should give the patient the employee’s specific work number and/or email assigned to biologic therapy patients to report as soon as possible when any adverse drug reaction is discovered, as it can be cleared by stopping the drug immediately and changing the patient to a different optimal therapy per the patient’s diagnosis.
